# Adherence to Mediterranean Diet and Soluble Klotho Level: The Value of Food Synergy in Aging

**DOI:** 10.3390/nu14193910

**Published:** 2022-09-21

**Authors:** Shou-En Wu, Ying-Jen Chen, Wei-Liang Chen

**Affiliations:** 1 Department of Dermatology, Tri-Service General Hospital, School of Medicine, National Defense Medical Center, Taipei 11490, Taiwan; 2 Department of Ophthalmology, Tri-Service General Hospital, School of Medicine, National Defense Medical Center, Taipei 11490, Taiwan; 3 Division of Family Medicine, Department of Family and Community Medicine, Tri-Service General Hospital, School of Medicine, National Defense Medical Center, Taipei 11490, Taiwan; 4 Division of Geriatric Medicine, Department of Family and Community Medicine, Tri-Service General Hospital, School of Medicine, National Defense Medical Center, Taipei 11490, Taiwan; 5 Department of Biochemistry, National Defense Medical Center, Taipei 11490, Taiwan

**Keywords:** soluble Klotho, Mediterranean diet, low-carbohydrate diet, low-fat diet, aging

## Abstract

Diets for healthy aging have long been an intriguing issue. The current study makes a head-to-head comparison of four dietary patterns and their associations with soluble Klotho (s-Klotho) levels, an aging-related marker. The dietary data of 7906 subjects were obtained from the National Health and Nutrition Examination Survey 2007–2016. Each participant was given a score or was grouped according to four dietary patterns, namely the Mediterranean adherence diet score (MDS), the low-carbohydrate-diet score, a low-fat diet, and a low-carbohydrate diet. Subsequently, the associations with s-Klotho were examined using linear regression analyses. In addition, we calculated the odds ratio (OR) for aging in different dietary patterns, taking the lowest quartile of s-Klotho as a reference for aging. The MDS was the only dietary pattern that revealed a relationship with s-Klotho levels. The positive association (β coefficient: 9.41, *p* < 0.001) remained significant when dividing the MDS into tertiles (Tertile 2: β coefficient: 36.87, *p* < 0.001; Tertile 3: β coefficient: 45.92, *p* < 0.001) and grouping participants into subsets by sex, age, and BMI. A lower OR for aging was observed in higher MDS groups (Tertile 2: OR = 0.86, *p* = 0.026; Tertile 3: OR = 0.77, *p* < 0.001). However, when analyzed separately, merely three out of nine components of the MDS, namely alcohol consumption (β coefficient: 42.54, *p* < 0.001), fruit (β coefficient: 11.59, *p* = 0.029), and dairy products (β coefficient: 8.55, *p* = 0.032), showed a significant association with s-Klotho. The Mediterranean diet adopts a food-based approach, which has the merit of valuing the complex interactions between foods and their constituents, and further brings benefits to healthy aging.

## 1. Introduction

Diet plays a crucial role in determining lifespan. Scientists have worked unceasingly on exploring a life-extending dietary pattern which grants health and wellness. Distinct dietary patterns show strengths in different aspects. For instance, the Mediterranean diet, which highlights monounsaturated fat (mainly virgin and extra-virgin olive oil) and a moderate intake of alcohol [1], has been demonstrated to reduce overall mortality [2], cardiovascular events [3], and the incidence of cancer [4]. Low-carbohydrate and low-fat diets have both been reported to bring positive effects to weight loss and a reduction in cardiovascular events [5,6]. In this research, we are committed to investigating a dietary pattern that brings the most benefits to healthy aging with the use of an anti-aging biomarker, soluble Klotho (s-Klotho) [7]. The Klotho gene and the Klotho protein it encodes have attracted scientists’ attention by virtue of the involvement of many pathways in the aging process. It regulates phosphate homeostasis [8], insulin-like growth factor 1 [9], and Wnt signaling pathways [10], while also protecting cells from oxidative stress and cell senescence [11]. s-Klotho is cleaved from the extracellular domain of membrane klotho and enters circulation to exert biological functions. With regard to the association between diet and s-Klotho, the number of studies is small. The main ones were published by the same group of researchers, who enrolled 74 middle-aged sedentary Spanish adults into their studies, revealing an inverse association with alcohol consumption [12], a weak positive association with dietary inflammation index [13], and a negative association in adherence to the Mediterranean diet, which disappeared after controlling for lean mass index [14]. Nevertheless, their studies were limited to small amounts of participants and reflected only certain age groups (45–65 years old).

To the best of our knowledge, this is the first study to make head-to head comparisons of different dietary patterns and aging, represented by the level of s-Klotho. The relatively large number of participants in our study represent the general population of the United States (US), and therefore may provide a more comprehensive view of the interactions between diet and healthy aging.

## 2. Materials and Methods

### 2.1. Study Design and Participants

The data of this study were retrieved from the National Health and Nutrition Examination Survey (NHANES) 2007–2016 [15]. The NHANES is a vital program administered by the National Center for Health Statistics (NCHS), which is part of the Centers for Disease Control and Prevention (CDC) that is responsible for the protection of public health and safety in the US. The NHANES continuously collects health and nutritional data of adults and children in the US and releases its dataset in two-year cycles with the aim of providing researchers with representative statistics of the general US population. The NHANES was conducted according to the guidelines of the Declaration of Helsinki, and study protocol was approved by the NCHS Institutional Review Board. It combined in-house interviews and physical examinations at a mobile examination center (MEC), and written informed consent was provided by each participant at the time of household interview.

**Figure 1:**
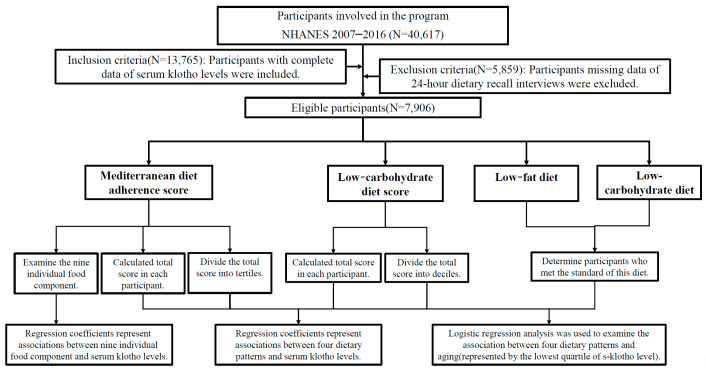
Flow chart of study population selection.

For the present study, the main research subject, s-Klotho, was available in the NHANES 2007–2016; hence, we focused on dietary datasets from those years. There were 40,617 individuals investigated in years 2007–2016, and 13,765 of them had data of s-Klotho levels. Afterwards, we inspected the data files of dietary recall interviews, and further excluded those with incomplete data (*n* = 5859). Finally, 7906 subjects were eligible for further analyses. A diagram depicting the design process of our study is provided in Figure 1.

### 2.2. Dietary Data

The dietary assessment of the NHANES was collected via a 24 h dietary recall method. Since 2002, two rounds of recall were carried out for comprehensive evaluation. Trained interviewers conducted the first dietary recall in-person at the MEC, and the second recall was scheduled 3–10 days later by telephone. This study adopted data from the first round of recall due to the some loss to follow-up in the second round. The dietary recalls in the NHANES were a portion of What We Eat in America, a primary project administered by the Food Surveys Research Group (FSRG), which is a part of the US Department of Agriculture, Agricultural Research Service. Other projects held by the FSRG were used in our study to examine the dietary information of our NHANES participants. The USDA Food and Nutrient Database for Dietary Studies (FNDDS) was used to convert individual food and beverages reported by participants into gram amounts or portion sizes in order to estimate their nutrient values [16]. Another specific database, the Food Patterns Equivalents Database (FPED), integrates individual food in the FNDDS and constructs 37 food and beverage groupings according to the 2015–2020 Dietary Guidelines for Americans recommendations [17]. We utilized FPED 2007–2008, 2009–2010, 2011–2012, 2013–2014, and 2015–2016 [18,19,20,21,22] for the calculation of the Mediterranean diet score.

### 2.3. Mediterranean Diet Adherence Score

The Mediterranean diet adherence score (MDS) in our study was calculated using a literature-based adherence score proposed by Sofi et al. [23]. It was composed of nine food components, which are fruit, vegetables, legumes, cereals, fish, meat and meat products, dairy products, alcohol, and olive oil. Each component was assigned a score of “0”, “1”, or “2”, and adding up all components gave a final MDS, ranging from 0 to 18. Higher scores reflect a better adherence to the Mediterranean dietary pattern, which are not fully equivalent to a higher intake of each component. For instance, a higher intake of meat and dairy products produces lower scores, while a moderate consumption of alcohol (1–2 alcohol unit/day) generates the highest score, followed by little (<1 alcohol unit/day) and excessive consumption (>2 alcohol unit/day). The categorization of 37 food components in the FPED into the MDS refers to a previous study that conducted a diet investigation in the NHANES 2011–2014 [24]. Some modifications were made to apply the units of measurement in the FPED to the scale constructed by Sofi et al. First, fruit, vegetables, and dairy products were calculated aggregately as cup equivalent (CE) intakes in the FPED. Modified fruit scores were <1 CE/d = “0”, ≥1 CE/d = “1”, and ≥2 Ces/d = “2”; modified vegetable scores were <0.5 CE/d = “0”, ≥0.5 CE/d = “1”, and ≥1 CE/d = “2”; and modified dairy product scores were >1.5 Ces/d CE = “0”, 1–1.5 Ces/d = “1”, and <1.5 Ces/d = “2”. Secondly, olive oil was presented in grams in the FPED; therefore, we transformed scores into <14 g/d = “0”, ≥14 g/d = “1”, and ≥28 g/d Ces/d = “2”. Other component scores complied with the original calculation proposed by Sofi et al. For further analysis, we also computed the 1st, 2nd, and 3rd tertile of the total MDS score. Details are shown in Table A1.

### 2.4. Low-Carbohydrate-Diet Score

The low-carbohydrate-diet (LCD) score was developed previously [25]. In brief, the three macronutrients (carbohydrate, protein, and fat) were calculated as a percentage (%) of total daily energy intake, and further divided into 11 strata. Scores were assigned from “0” to “10” to each stratum, but in a different ordered sequence. In the lowest stratum of intake, protein and fat scored “0”, while carbohydrate scored “10”. In other words, the scores for protein and fat were ranked in an ascending order according to the percentage of intake, while carbohydrate was ranked in a descending order. Summing up the scores of the three macronutrients gave an overall score ranging from “0” to “30”, which was the final LCD score. For further analysis, we also computed the 1st, 5th, and 10th decile of the total LCD score. Details are shown in Table A1.

### 2.5. Low-Fat Diet and Low-Carbohydrate Diet

The low-fat diet was defined following the American Heart Association Dietary Guidelines [26], which recommended that <30% of total daily calorie intake come from fats. The low-carbohydrate (low-carb) diet in our study adopted the definition from the American Academy of Family Physicians [27], which specified a low-carb diet as containing <20% of total daily calorie intake from carbohydrates. Details are shown in Table A1.

### 2.6. Measurement of s-Klotho Levels

According to the NHANES Laboratory/Medical Technologists Procedures Manual [28], s-Klotho levels were examined using the IBL ELISA kit (Immuno-Biological Laboratories, Gunma, Japan). Duplicate tests of the sample yielded 3.8% and 3.4% for the inter-assay coefficients of variation. The reference range for s-Klotho was 285.8 to 1638.6 pg/mL.

### 2.7. Covariates

Self-reported demographic information was obtained in household interviews. The computation of body mass index used the formula: kg/m^2^ (weight in kilograms, height in meters squared). A positive smoking history was determined by smoking at least 100 cigarettes in life. The subjects’ history of medical conditions, including congestive heart failure, coronary heart disease, angina, diabetes, and hypertension were acquired by asking the participants if they were ever told by doctors or other health professional that they had the aforementioned condition.

### 2.8. Statistical Analyses

The software used for analysis was SPSS (IBM Corp. Released 2013. IBM SPSS Statistics for Windows, Version 22.0. Armonk, NY, USA: IBM Corp.). Linear regression analyses were used to examine the association, and β coefficients provided an estimate of the strength of the relationship. Odds ratios (ORs) were calculated by logistic regression analyses. In the present study, they evaluated the association between exposure (scoring in different dietary pattern) and outcome (aging, represented by the lowest quartile of s-Klotho levels). In all analyses, results were regarded as significant when *p* values were less than 0.05. Three adjustment models were provided in each analysis: Model 1 = unadjusted; Model 2 = adjusted for adjusted for age, sex, body mass index, and race/ethnicity; Model 3 = Model 2 + adjusted for history of angina, congestive heart failure, coronary heart disease, diabetes mellitus, hypertension, and smoking status. Model 2 reckoned with the effect of general health conditions, and Model 3 further considered the impact of common aging-related diseases.

## 3. Results

### 3.1. Characteristics of Participants

Demographic, laboratory, and dietary information are presented in Table 1. The mean age of the participants was 57.77 ± 10.92 years old. The mean MDS and LCD scores were 6.72 ± 2.12 and 14.98 ± 7.19, respectively. The mean percentage of daily calorie intake from carbohydrates, protein, and fats were 49%, 16%, and 34%, respectively.

### 3.2. Associations between Different Dietary Patterns and s-Klotho Levels

We observed a positive association between the MDS and s-Klotho levels, as shown in Table 2. When calculated as a total score, the β coefficient (95% CI) was 9.41 (6.08–12.74), indicating a 9.41 pg/mL rise in s-Klotho levels per 1-score increase in the MDS (*p* < 0.001) in the unadjusted model. The adjusted model also revealed a significant positive correlation (both *p* < 0.001). When calculated as scores divided into tertiles, the higher tertiles revealed a stronger association with s-Klotho levels compared to the lowest tertile (Tertile 2: β coefficient (95% CI) = 36.87(19.17–54.57), *p* < 0.001; Tertile 3: β coefficient (95% CI) = 45.92 (28.01–63.83), *p* < 0.001). By comparison, the other three dietary patterns did not reveal a significant relationship with s-Klotho levels.

### 3.3. Association between Different Dietary Patterns and Aging

s-Klotho levels were demonstrated to be inversely associated with age in previous studies [29,30]. Accordingly, we reason that the lowest quartile of s-Klotho levels correlates with a higher likelihood of aging. As shown in Table 3, logistic regression analyses were used to calculate the ORs for aging (represented by quartile 1 of s-Klotho) in different dietary scores. In the MDS and the LCD score, the reference groups were the lowest tertile and decile, while in the low-fat diet and the low-carb diet, ORs were compared to those who did not meet the standards mentioned in the previous section. The results of the MDS revealed that ORs gradually decreased from tertile 2 to 3, and both were less than 1, indicating protective effects from aging in higher MDS groups (Tertile 2 of MDS: OR = 0.86, *p* = 0.026; Tertile 3 of MDS: OR = 0.77, *p* < 0.001 compared to Tertile 1). The other three dietary patterns did not disclose significant results.

### 3.4. Associations across Tertiles of MDS and s-Klotho Levels in Subgroups

Owing to the significant relationship revealed in the MDS, either calculated as the total score or in tertiles, we were interested in whether the correlation was still present in specific groups. In Table 4, we sorted participants by sex, age, and BMI and examined the relationship by linear regression analyses. In men, the β coefficients were higher in tertile 3 (β coefficient: 43.48, 95% CI = 20.39–66.56, *p* < 0.001) than tertile 2 (β coefficient: 26.62, 95% CI = 4.18–49.05, *p* = 0.020). Meanwhile, in women, the β coefficients were of a similar value in tertile 2 (β coefficient: 41.01, 95% CI = 13.34–68.69, *p* = 0.004) and 3 (β coefficient: 40.28, 95% CI = 12.63–67.94, *p* = 0.004). In addition, a stronger relationship with s-Klotho (presented as a higher β coefficient value) appeared in women compared to men. When sorted by age, older adults (≥ 65 years old) revealed a stronger relationship with s-Klotho. In the unadjusted model, the younger group had a β coefficient = 33.44 and 48.38 in tertile 2 and 3, respectively, while the older group had a β coefficient = 55.30 and 55.75 in tertile 2 and 3, respectively. When sorted by BMI, participants with a greater BMI (≥30) presented a stronger relationship with s-Klotho. In the unadjusted model, participants with a lower BMI had a β coefficient = 29.98 and 40.56 in tertile 2 and 3, respectively, while the older group had a β coefficient = 46.82 and 53.69 in tertile 2 and 3, respectively. It is noteworthy that all subgroups revealed statistically significant results.

### 3.5. Associations between Nine Individual Food Components of MDS and s-Klotho Levels

**Figure 2:**
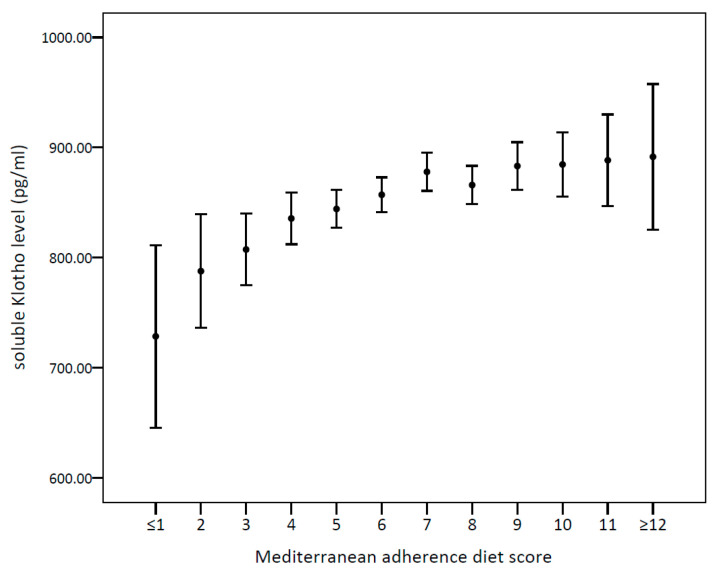
A line chart showing the change of mean s-Klotho levels in Mediterranean diet adherence score from the lowest (score ≤ 1) to the highest (score ≥ 12).

We further investigated in nine individual food components of the MDS to examine which ones showed a significant association with s-Klotho. Figure 2 presents a forest plot of the β coefficient, which discloses the degree of change in s-Klotho levels for each 1-score increase in individual components. Among the nine, only three components revealed significant results. The most notable one was alcohol consumption, with a β coefficient of 42.54 (95% CI = 32.09–52.99, *p* < 0.001). The other two were fruit (β coefficient: 11.59, 95% CI = 1.21–21.97, *p* = 0.029) and dairy products (β coefficient: 8.55, 95% CI = 0.73–16.37, *p* = 0.032).

**Figure 3:**
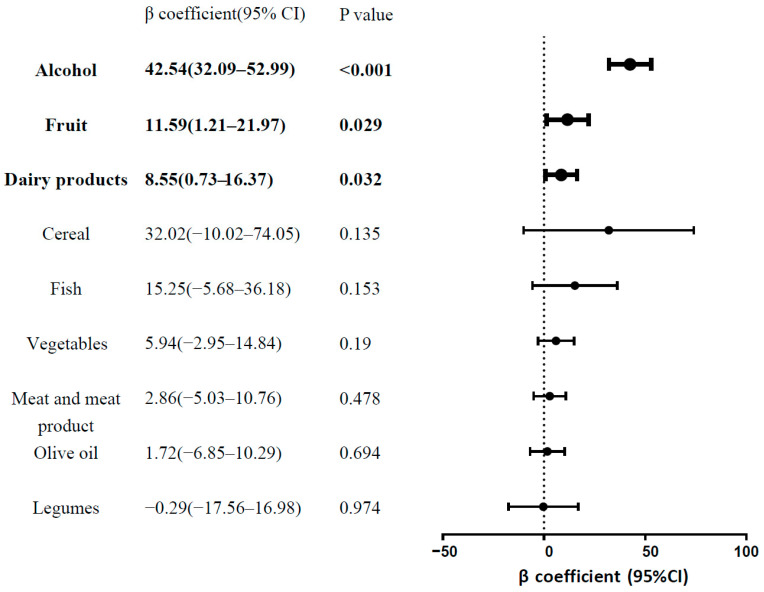
Forest plot of β coefficients presenting the association between nine components in the Mediterranean diet adherence score and s-Klotho levels as outcome. The β coefficient discloses the degree of change in s-Klotho levels for each 1-score increase in an individual component. Components that revealed significant associations are highlighted in bold.

Owing to the result that most components did not reveal a significant relationship with s-Klotho when viewed separately, we calculated the change of mean s-Klotho levels in the total MDS, as shown in Figure 3. We combined the extreme maximum and minimum scores due to the scant number in these groups (scores 0 and 1 combined into ≤1, and scores 12–18 combined into ≥12). A steady upward trend can be observed from the lowest to the highest MDS group (*p* < 0.001), demonstrating that the positive association between the MDS and s-Klotho levels is revealed only when viewed as a whole.

## 4. Discussion

The current study highlights the positive association between the MDS and s-Klotho levels, and the association remains significant in all subgroups, including men and women, the young and the old, and the obese and the non-obese. In addition, on the assumption that the lowest quartile of s-Klotho correlates to aging, a higher MDS indicates protective effects against aging. On the other hand, the other three dietary pattern of interest, namely the LCD, low-fat diet, and low-carb diet failed to show any association with s-Klotho or aging.

The Mediterranean diet and healthy aging have been widely discussed in the literature. The first direct evidence regarding lifespan was demonstrated in a study collected in Greek villages, showing a one-unit increase in diet score related to a 17% reduction in overall mortality [31]. Subsequently, studies conducted outside the Mediterranean region, where people generally followed the spirit of the Mediterranean diet but did not rigorously follow the traditional version, also revealed a significantly lower mortality risk [32,33]. Mechanisms that have been validated as a contribution to its protective property include its antioxidant and anti-inflammatory capacity [34], lipid-lowering effects [35], and improvement in insulin sensitivity [36].

Evidence for the relationship between carbohydrates and lifespan is conflicting in humans. A low-carbohydrate diet is reported to bring benefits to weight loss [5], coronary heart disease [37], and diabetes mellitus [38], but several studies showed increased mortality in adults consuming low-carbohydrate–high-protein diets [39,40]. One study, however, showed no clear association with mortality [41]. Other studies in different organisms targeting carbohydrates and lifespan revealed varied results. In *C. elegans*, the restriction of glucose availability was proved to extend lifespan [42]. On the contrary, strategies of increasing the carbohydrate-to-protein ratio were demonstrated to maximize life expectancy in Drosophila [43] and mice [44]. With regard to dietary lipids, a low-fat diet contributes to weight loss [45], but recent evidence points out the importance of collaboratively controlling glycemic load [46] and total calorie intake [47]. In addition, specific lipids induce heterogeneous effects. For instance, a polyunsaturated fatty acid-supplemented diet extends the lifespan in *C. elegans* [48] but shortens the lifespan in mice [49]. Researchers have proposed the possibility that fatty acid compositions on cellular membranes are essential factors in the determination of longevity [50] rather than dietary lipids. Our study, however, found no significant association with s-Klotho levels in either the LCD or low-carb diet or low-fat diet. Discordant results may be attributed to the complex cellular mechanisms linking diet and aging, and centering on a portion of a single macronutrient could not draw solid conclusions.

The findings of the association between individual food components of the MDS and s-Klotho levels echo previous studies investigating the association with all-cause mortality. A meta-analysis that summarized the results from 16 Mediterranean diet studies, with respect to longevity, reported that the excess relative risk was the highest for moderate alcohol intake (−14%), followed by fruit (−12%) [51]. The summary relative risk (RR) in association with all-cause mortality also revealed that the most notable inverse association was with alcohol intake (RR = 0.86) and fruit (RR = 0.88). By contrast, other components revealed minimal association (RR ranged from 0.94–1.07), or even null association, for legumes (RR = 1.00) and fish intake (RR = 1.01). Another review article compared the potential benefits of the Mediterranean diet or its components toward cardiovascular disease [52]. They concluded that, despite each component yielding protective effects to a certain extent, none of them proved to be as beneficial as the whole Mediterranean diet for the primary and secondary prevention of cardiovascular disease. Our study, although with a different research outcome, revealed consistent findings with merely three components presenting significant associations. The favorable impacts brought by the Mediterranean diet can be well explained by the concept of “food synergy”, which emphasizes the stronger health benefits lying in a total diet rather than individual foods or food constituents [53,54]. Several advantages have been proposed. A synergetic dietary pattern serves a buffer effect in which each constituent is balanced, rather than an excessive intake of a single one. Furthermore, the physical and chemical interactions between various foods and with the body system, which is specifically termed ”the food matrix”, are essential in overall health [55].

The concept of food synergy not only delineates the findings that the Mediterranean diet is more powerful as a whole, but also illustrates the results that the Mediterranean diet is the only diet in our study that correlates with s-Klotho levels. The feature that distinguishes the Mediterranean diet from the other three is that it is a food-based approach rather than a nutrient-based approach, which has been proposed to be a fundamental strategy that gives the full picture of the complex metabolic interrelations between foods. Scientists argue that, despite the fact that identifying single food or nutrient provides a better understanding in several cases, a comprehensive knowledge of food and its relationship with general health should be underpinned by an integrated model, the “whole food-oriented research” [56] or ”nutritional geometry” [57]. The Mediterranean diet, being a pyramid-shaped eating pattern that comprises a large base of plant foods, a middle layer of fish, poultry, and dairy products, and an apex of red meat products and sweets, has the merit of stressing the assembly of foods and an adequate amount of each. In comparison to other macronutrient-based diets, it provides a blended diet that values the constituents and nutrients of each food, whether abundant or scarce, playing a vital role in the multidimensional framework of nutrition. This accounts for the main reason why the Mediterranean diet shows significant results in all analyses.

Another issue that deserves attention is how the ‘type’ and ‘amount’ of alcohol have an influence on Klotho and ageing. In the Mediterranean diet, the overall intake is more emphasized, with studies supporting the notion that controlling the amount, irrespective of the beverage source, brings protective effects [58,59]. However, there is evidence illustrating that red wine may somehow show a more potent capability of cardiovascular protection [60,61]. Others have argued that this phenomenon can observed in countries that habitually consume wine rather than other alcoholic beverages, terming it the ‘French paradox’ [62,63]. To date, these observational results remain an issue for debate. From the present study, we can only tell that consuming alcohol in moderation correlates with higher s-Klotho levels, but a conclusion regarding the differences between types of alcohol cannot be drawn due to a lack of corresponding data.

Some limitations in the present study should be acknowledged. First, some modifications were made to the calculation of the MDS due to different units of measurements in the original version of the MDS by Sofi et al. [23] and the accessible dietary data in the NHANES. One previous NHANES study on the Mediterranean diet [24] encountered the same predicament, and we referred to their conversion methods in the hopes of minimizing the statistical error. Secondly, causal inference in cross-sectional studies, e.g., the NHANES, is hard to approach. Associations could be observed between exposures and outcomes, but the direction of cause and effect within is difficult to determine in practice. Further research is needed to verify the underlying cause of the association.

## 5. Conclusions

In the present study, the MDS revealed a positive association with s-Klotho levels, an anti-aging biomarker, while the other three dietary patterns, namely the LCD, low-fat diet, and low-carb diet showed null association. The association remained significant when participants were grouped into subsets by sex, age, and BMI. In addition, the association disappeared in six out of nine components in the MDS when viewed separately. The Mediterranean diet differs from the other three by offering a combination of foods instead of stressing the individual macronutrients, which embodies the spirit of “food synergy” that values the complex interactions between food and its constituents. Future studies are warranted to investigate how diets influence s-Klotho levels and other aging-related biomarkers.

## Figures and Tables

**Table 1:** Characteristics of participants.

Characteristics	Total Participants (*n* = 7906)
^2^ Continuous variables	
Age (years)	57.77 ± 10.92
^1^ BMI (kg/m^2^)	29.67 ± 6.54
Klotho (pg/mL)	861.17 ± 311.55
Mediterranean diet score	6.72 ± 2.12
Low-carbohydrate diet score	14.98 ± 7.19
Daily carbohydrate intake (gm)/Percentage of calories from carbohydrate	245.50 ± 116.27 (49%)
Daily protein intake (gm)/Percentage of calories from protein	78.67 ± 39.30 (16%)
Daily fat intake (gm)/Percentage of calories from fat	76.10 ± 43.96 (34%)
Daily calorie intake (kcal)	2020.97 ± 923.62
^3^ Categorical variables	
Sex	
Men	3910 (49.5%)
Women	3996 (50.5%)
Race	
Mexican American	1225 (15.5%)
Other Hispanic	855 (10.8%)
Non-Hispanic White	3704 (46.9%)
Non-Hispanic Black	1619 (20.5%)
Other race, including multi-racial	503 (6.4%)
History of congestive heart failure	312 (3.9%)
History of coronary heart disease	392 (5%)
History of angina	254 (3.2%)
History of diabetes mellitus	6705 (84.8%)
History of hypertension	5433 (68.7%)
Smoking history	3987 (50.4%)

^1^ BMI: body mass index. ^2^ Values in the continuous variables were expressed as mean and standard deviation. ^3^ Values in the categorical variables were expressed in number and percentage (%).

**Table 2:** Linear regression analyses for the associations between different dietary patterns and serum klotho levels.

		^1^ MDS				^2^ LCD Score				Low-Fat Diet	^3^ Low-Carb Diet
		Total Score	Tertile			Total Score	Decile				
			1	2	3		1	5	10		
Model 1	β coefficient (95% CI)	9.41 (6.08, 12.74)	Ref.	36.87 (19.17, 54.57)	45.92 (28.01, 63.83)	−0.366 (−1.355, 0.623)	Ref.	1.45 (−19.82, 22.72)	−6.32 (−28.30, 15.67)	−2.16 (−16.97, 12.66)	−96.08 (−202.49, 10.32)
	*p* value	<0.001	Ref.	<0.001	<0.001	0.468	Ref.	0.894	0.573	0.775	0.077
Model 2	β coefficient (95% CI)	9.62 (6.29, 12.95)	Ref.	37.38 (19.76, 55.01)	46.79 (28.87, 64.72)	−0.116 (−1.106, 0.873)	Ref.	5.24 (−15.91, 26.40)	−1.16 (−14.58, 31.66)	−2.58 (−17.37, 12.21)	−95.37 (−201.14, 10.39)
	*p* value	<0.001	Ref.	<0.001	<0.001	0.818	Ref.	0.627	0.917	0.732	0.077
Model 3	β coefficient (95% CI)	9.01 (5.67, 12.34)	Ref.	35.92 (18.33, 53.51)	43.42 (25.50, 61.34)	−0.200 (−1.188, 0.788)	Ref.	3.76 (−17.34, 24.85)	−2.56 (−24.48, 19.35)	−1.80 (−16.56, 12.97)	−103.54 (−208.98, 1.89)
	*p* value	<0.001	Ref.	<0.001	<0.001	0.691	Ref.	0.727	0.819	0.811	0.054

^1^ MDS: Mediterranean diet adherence score; ^2^ LCD score: low-carbohydrate-diet score; ^3^ Low-carb diet: low-carbohydrate diet. Model 1 = unadjusted. Model 2 = adjusted for age, sex, body mass index, and race/ethnicity. Model 3 = Model 2 + adjusted for history of angina, congestive heart failure, coronary heart disease, diabetes mellitus, hypertension, and smoking history.

**Table 3:** Odds ratios (95% confidence intervals) for lowest quartile of serum Klotho levels (represents aging) in various dietary patterns.

		^1^ MDS			^2^ LCD Score			Low-Fat Diet	^3^ Low-Carb Diet
		Tertile			Decile				
		1	2	3	1	5	10		
Model 1	OR (95% CI)	Ref.	0.86 (0.76, 0.98)	0.77 (0.68, 0.88)	Ref.	0.97 (0.83, 1.13)	0.96 (0.82, 1.13)	1.01 (0.91, 1.13)	1.50 (0.73, 3.11)
	*p* value	Ref.	0.026	<0.001	Ref.	0.653	0.648	0.867	0.271
Model 2	OR (95% CI)	Ref.	0.85 (0.75, 0.97)	0.76 (0.66, 0.86)	Ref.	0.95 (0.81, 1.11)	0.94 (0.80, 1.11)	1.01 (0.91, 1.13)	1.52 (0.73, 3.15)
	*p* value	Ref.	0.017	<0.001	Ref.	0.512	0.478	0.929	0.260
Model 3	OR (95% CI)	Ref.	0.86 (0.75, 0.98)	0.77 (0.67, 0.88)	Ref.	0.96 (0.82, 1.12)	0.95 (0.81, 1.12)	1.01 (0.89, 1.12)	1.58 (0.76, 3.29)
	*p* value	Ref.	0.024	<0.001	Ref.	0.956	0.949	0.933	0.217

^1^ MDS: Mediterranean diet adherence score; ^2^ LCD score: low-carbohydrate-diet score; ^3^ Low-carb diet: low-carbohydrate diet. Model 1 = unadjusted. Model 2 = adjusted for age, sex, body mass index, and race/ethnicity. Model 3 = Model 2 + adjusted for history of angina, congestive heart failure, coronary heart disease, diabetes mellitus, hypertension, and smoking history.

**Table 4:** Associations across tertiles of Mediterranean diet score and serum Klotho levels in subgroups.

		Tertile 1 of ^1^ MDS	Tertile 2 of MDS	Tertile 3 of MDS
Sorted by sex				
Men				
Model 1	β coefficient (95% CI)	Ref.	26.62 (4.18, 49.05)	43.48 (20.39, 66.56)
	*p* value	Ref.	0.020	<0.001
Model 2	β coefficient (95% CI)	Ref.	29.73 (7.34, 52.13)	49.59 (26.45, 72.73)
	*p* value	Ref.	0.009	<0.001
Model 3	β coefficient (95% CI)	Ref.	27.81 (5.47, 50.16)	45.31 (22.16, 68.47)
	*p* value	Ref.	0.015	<0.001
Women				
Model 1	β coefficient (95% CI)	Ref.	41.01 (13.34, 68.69)	40.28 (12.63, 67.94)
	*p* value	Ref.	0.004	0.004
Model 2	β coefficient (95% CI)	Ref.	45.34 (17.79, 72.89)	45.01 (17.43, 72.59)
	*p* value	Ref.	0.001	0.001
Model 3	β coefficient (95% CI)	Ref.	43.22 (15.75, 70.69)	41.23 (13.67, 68.79)
	*p* value	Ref.	0.002	0.003
Sorted by age				
<65 years old				
Model 1	β coefficient (95% CI)	Ref.	33.44 (12.83, 54.05)	48.38 (27.29, 69.48)
	*p* value	Ref.	0.001	<0.001
Model 2	β coefficient (95% CI)	Ref.	31.58 (11.01, 52.15)	45.22 (24.09, 66.35)
	*p* value	Ref.	0.003	<0.001
Model 3	β coefficient (95% CI)	Ref.	30.59 (10.09, 51.09)	40.19 (19.07, 61.32)
	*p* value	Ref.	0.003	<0.001
≥65 years old				
Model 1	β coefficient (95% CI)	Ref.	55.30 (20.99, 89.62)	55.75 (21.85, 89.65)
	*p* value	Ref.	0.002	0.001
Model 2	β coefficient (95% CI)	Ref.	53.87 (19.54, 88.21)	52.40 (18.43, 86.38)
	*p* value	Ref.	0.002	0.003
Model 3	β coefficient (95% CI)	Ref.	51.03 (16.68, 85.38)	49.61 (15.54, 83.68)
	*p* value	Ref.	0.004	0.004
Sorted by BMI				
BMI < 30				
Model 1	β coefficient (95% CI)	Ref.	29.98 (6.66, 53.32)	40.56 (16.93, 64.19)
	*p* value	Ref.	0.012	0.001
Model 2	β coefficient (95% CI)	Ref.	31.09 (7.86, 54.31)	40.98 (17.31, 64.66)
	*p* value	Ref.	0.009	0.001
Model 3	β coefficient (95% CI)	Ref.	27.93 (4.74, 51.11)	36.67 (12.97, 60.38)
	*p* value	Ref.	0.018	0.002
BMI ≥ 30				
Model 1	β coefficient (95% CI)	Ref.	46.82 (19.67, 73.97)	53.69 (26.25, 81.13)
	*p* value	Ref.	0.001	<0.001
Model 2	β coefficient (95% CI)	Ref.	45.52 (18.42, 72.61)	54.14 (26.68, 81.59)
	*p* value	Ref.	0.001	<0.001
Model 3	β coefficient (95% CI)	Ref.	46.29 (19.27, 73.31)	51.86 (24.46, 79.26)
	*p* value	Ref.	0.001	<0.001

^1^ MDS: Mediterranean diet adherence score. Model 1 = unadjusted. Model 2 = adjusted for age, sex, body mass index, and race/ethnicity. Model 3 = Model 2 + adjusted for history of angina, congestive heart failure, coronary heart disease, diabetes mellitus, hypertension, and smoking history.

## Data Availability

The datasets generated and analyzed during the current study are publicly available from the NHANES website. (https://wwwn.cdc.gov/nchs/nhanes/nhanes3/default.aspx, accessed on 12 July 2022).

## Appendix A

**Table A1:** The definition and calculation method of four dietary patterns in the present study.

	Mediterranean Adherence Diet Score (MDS)				Low-Carbohydrate-Diet Score (LCD)			Low-Fat Diet	Low-Carbohydrate Diet
Definition	A score ranging from 0–18 by adding up nine components.				A score ranging from 0–30 by adding up individual scores of three macronutrients, carbohydrate, protein, and fat.			<30% of total daily calorie intake from fat	<20% of total daily calorie intake from carbohydrate
	Each component is assigned a score of “0”, “1”, or “2” according to the amount of intake.				Each macronutrient was calculated as a percentage (%) of total daily energy intake, and further divided into 11 strata.				
Calculation method	Scores	0	1	2	Scores	Carbohydrate	Protein/Fat		
	Fruit	<1 CE/d	≥1 CE/d	≥2 CE/d	0	11th stratum	1st stratum		
	Vegetables	<0.5 CE/d	≥0.5 CE/d	≥1 CE/d	1	10th stratum	2nd stratum		
	1 portion = 70 g = 2.5 oz				2	9th stratum	3rd stratum		
	Cereals	<4.6 oz/d	4.6–6.9 oz/d	>6.9 oz/d	3	8th stratum	4th stratum		
	1 portion = 130 g = 4.6 oz				4	7th stratum	5th stratum		
	Fish	<3.5 oz/wk	3.5–8.75 oz/wk	>8.75 oz/wk	5	6th stratum	6th stratum		
	1 portion = 100 g = 3.5 oz				6	5th stratum	7th stratum		
	Meat product	>4.2 oz/d	2.8–4.2 oz/d	<2.8 oz/d	7	4th stratum	8th stratum		
	1 portion = 80 g = 2.8 oz				8	3rd stratum	9th stratum		
	Dairy products	>1.5 CE/d	1–1.5 CE/d	<1 CE/d	9	2nd stratum	10th stratum		
	Alcohol (mL/day)	>2 AU/d	<1 AU/d	1–2 AU/d	10	1st stratum	11th stratum		
	1 AU = 12 g								
	Olive oil (gm)	<14 g/d	≥14 g/d	≥28 g/d					
	CE = cup equivalent intakes; d = day; wk = week; gm = gram; mL = milliliter; oz = ounce								
References	Previous literature by Sofi et al. [23]				Previous literature by Halton et al. [25]			American Heart Association Dietary Guidelines [5]	American Academy of Family Physicians [26]
